# Occupational health and safety in the wake of COVID-19: insights from India's workforce

**DOI:** 10.3389/fpubh.2026.1807940

**Published:** 2026-06-09

**Authors:** Nikita Birhman, Gitika Kharkwal, Sudipto Roy, Navsin Shaikh, Poonam Sharma Velamuri, Hem Lata, Abhishweta Saxena, Aanchal Satija, Kh Jitenkumar Singh, Taruna Madan, Jugal Kishore, Prakash Kanti, Tanu Anand

**Affiliations:** 1 Division of Development Research, ICMR Hqrs, New Delhi, India; 2 Division of Biological Sciences, ICMR-NIOH, Ahmedabad, India; 3 Academy of Scientific and Innovative Research, Ghaziabad, Uttar Pradesh, India; 4 ICMR-RMRC, Dibrugarh, India; 5 Discovery Division, ICMR Hqrs, New Delhi, India; 6 DHR, New Delhi, India; 7 Data Centre ICMR Hqrs., New Delhi, India; 8 Department of Community Medicine, Vardhman Mahavir Medical College & Safdarjung Hospital, New Delhi, India

**Keywords:** COVID-19 pandemic, infection control, mental health, occupational health and safety (OHS), personal protective equipment (PPE), workplace transmission

## Abstract

**Introduction:**

Occupational health and safety (OHS) practices witnessed profound changes worldwide during the COVID-19 pandemic. The pandemic exposed critical vulnerabilities in workplace preparedness, particularly among workers in high-exposure and essential service sectors.

**Methods:**

A systematic review was conducted following PRISMA guidelines using PubMed, Google Scholar, DOAJ, ResearchGate, and gray literature sources. Studies published between January 2020 and December 2024 focusing on occupational exposure, workplace transmission, OHS guidelines, and workforce health during COVID-19 were included.

**Results:**

Healthcare workers experienced the highest occupational risk due to sustained patient contact and aerosol-generating procedures, accompanied by significant psychological distress and burnout. Essential workers, manufacturing sectors, retail workers, educators, and informal sector workers also faced substantial occupational and socioeconomic challenges. Workplace interventions including engineering controls, administrative controls, PPE use, vaccination strategies, and digital surveillance tools reduced workplace transmission. Remote-enabled sectors demonstrated lower infection risk but increased mental health concerns.

**Discussion:**

The COVID-19 pandemic transformed traditional OHS frameworks by integrating infectious disease preparedness, vaccination strategies, mental health support, and workplace surveillance into occupational safety systems. The findings emphasize the need for integrated, adaptive, and equitable OHS frameworks aligned with public health preparedness to strengthen workforce resilience against future pandemics.

## 1 Introduction

Occupational health and safety (OHS) practices were affected on an unprecedented global scale by the COVID-19 pandemic (1). The International Labor Organization (ILO)/World Health Organization (WHO) (1950) OHS as the multidisciplinary field of promoting and maintaining the maximum level of social, mental, and physical wellbeing for workers in all occupations. It focuses on reducing illnesses, accidents, and injuries related to the workplace while modifying the workplace to accommodate employees physical and mental capacities (2). The patterns of COVID-19 transmission highlighted occupational settings as potential and critical hotspots for viral spread (3). Jobs requiring close physical interaction, such as HCWs, frontline personnel, and service industry employees posed a higher risk of exposure to Severe Acute Respiratory Syndrome-Coronavirus-2 (SARS-CoV-2). Thus, workplace dissemination of SARS-CoV-2 infection was identified as a major contributor to the spread of the virus, with significant outbreaks reported in high-risk occupational environments including healthcare centers, poultry/meat manufacturing plants, and detention centers (4). Other sectors, including IT, education, tourism, transport, and production, also experienced considerable operational disruptions and workforce challenges (5, 6). Further, factors such as limited access to personal protective equipment (PPE), overcrowded workspaces, and poor ventilation exacerbated the vulnerability of certain occupational groups to infection. In addition, fear of isolation from their families and lack of adequate support for daily needs also led many such workers to conceal their symptoms which further complicated the efforts to control transmission (7, 8). Considering COVID-19 as an occupationally acquired infection expanded beyond traditional high-risk sectors, with evidence indicating occupational origin of infections extended across all economic activities and occupations (9). This association between occupation and COVID-19 infection garnered significant recognition due to its implications for public health policies, workplace safety measures, and risk assessment strategies. In response, evidence-based infection control measures—including engineering controls (improved ventilation and physical barriers), administrative strategies (rotational shifts, remote work, screening, contact tracing and worker training), safe work practices, and appropriate use of PPE—were widely implemented to reduce workplace transmission (4, 10, 11). In addition to infection control measures, occupational health frameworks recognize multiple categories of workplace hazards, including biological, chemical, physical, environmental/mechanical, and psychosocial risks, all of which may influence worker health and safety (12). For airborne infectious diseases such as COVID-19, engineering controls—such as improved ventilation systems and high-efficiency particulate air (HEPA) filtration—play a critical role in reducing the concentration of infectious aerosols in indoor workplaces. As emphasized by the National Institute for Occupational Safety and Health (NIOSH), these engineering interventions represent higher-level control measures that are generally more effective than relying solely on individual protective practices. This structured hierarchy-based approach prioritizes elimination and engineering solutions, followed by administrative controls and personal protective equipment (PPE), to minimize occupational hazards and reduce workplace transmission (12). Application of this framework contributed significantly to limiting occupational spread of COVID-19 and preventing further community transmission.

Beyond immediate infection control, the COVID-19 pandemic reshaped OHS frameworks worldwide. Traditional OHS approaches, which largely focused on physical and chemical hazards, expanded to incorporate infectious disease preparedness, workforce surveillance, vaccination, mental health support, and digital health tools. COVID-19 led to the recognition of infectious disease exposure as an occupational risk across diverse sectors, prompting the development of adaptive, risk-based workplace guidelines by global and national agencies (13, 14). In India, this transformation was reflected through comprehensive workplace advisories, prioritization of vaccination for high-risk occupational groups, integration of public health measures into routine workplace practices, and strengthened protections for vulnerable workers, including those in informal and migrant sectors. These shifts underscore the emergence of a more integrated, resilient OHS paradigm capable of responding to future public health emergencies (15).

As of November 2024, there were 776,841,264 confirmed cases and 7,075,468 reported deaths globally (16). As of May 26, 2025, India identified new COVID-19 subvariants NB.1.8.1 and LF.7, both derivatives of the JN.1 Omicron lineage, with cases reported in Tamil Nadu, Gujarat, and Uttar Pradesh. Milder variants may enhance transmission by increasing the mobility of infected individuals, thereby facilitating spread to higher-risk populations. This resurgence underscores the need for reinforced OHS measures within India's workforce, including the implementation of preventive protocols such as respirator use in occupational settings, hand hygiene, and physical distancing, particularly in high-density work environments. Additionally, promoting booster vaccinations among employees can serve as a proactive strategy to mitigate potential outbreaks, thereby safeguarding both public health and economic productivity (17).

In India, a country with a vast and diverse occupational workforce and deep-rooted socioeconomic disparities, the interplay between COVID-19 exposure and occupations presents a unique set of challenges. Understanding this relationship is vital for designing targeted, context-specific interventions and minimizing workplace-related transmission. Although several studies have examined workplace exposure and transmission of COVID-19 globally, a comprehensive synthesis focusing on sector-specific risks, workplace mitigation strategies, and occupational health initiatives in India remains limited. Johannes V. Gross et al. reported that HCWs experienced substantially higher exposure to SARS-CoV-2 and emphasized the importance of comprehensive preventive strategies across occupational settings (11). Similarly, guidance from the WHO highlighted the need to strengthen OHS measures for health workers during the pandemic (18). In addition, Blustein et al. (19) demonstrated that COVID-19 significantly disrupted employment conditions and workplace safety worldwide, underscoring the broader occupational implications of the pandemic. Evidence from India by Khasne et al. (20) further revealed considerable burnout and psychological distress among IT during this period. Collectively, these studies highlight the significant occupational risks associated with COVID-19 and reinforce the need for a comprehensive synthesis of evidence on OHS during the pandemic, particularly in the context of the Indian workforce as well as globally.

Therefore, we conducted a systematic review of Indian and global evidence to examine the differential occupational risks of COVID-19 across diverse employment sectors, assess the effectiveness of workplace prevention and control measures, and synthesize lessons from public OHS responses implemented during the pandemic. This systematic review provides an integrated perspective on how the COVID-19 pandemic influenced occupational health policies and workplace practices, particularly within the context of India's diverse workforce. The insights gained aim to inform the development of more resilient, equitable, and adaptive occupational health frameworks capable of addressing future pandemics effectively.

## 2 Methodology

**Figure 1:**
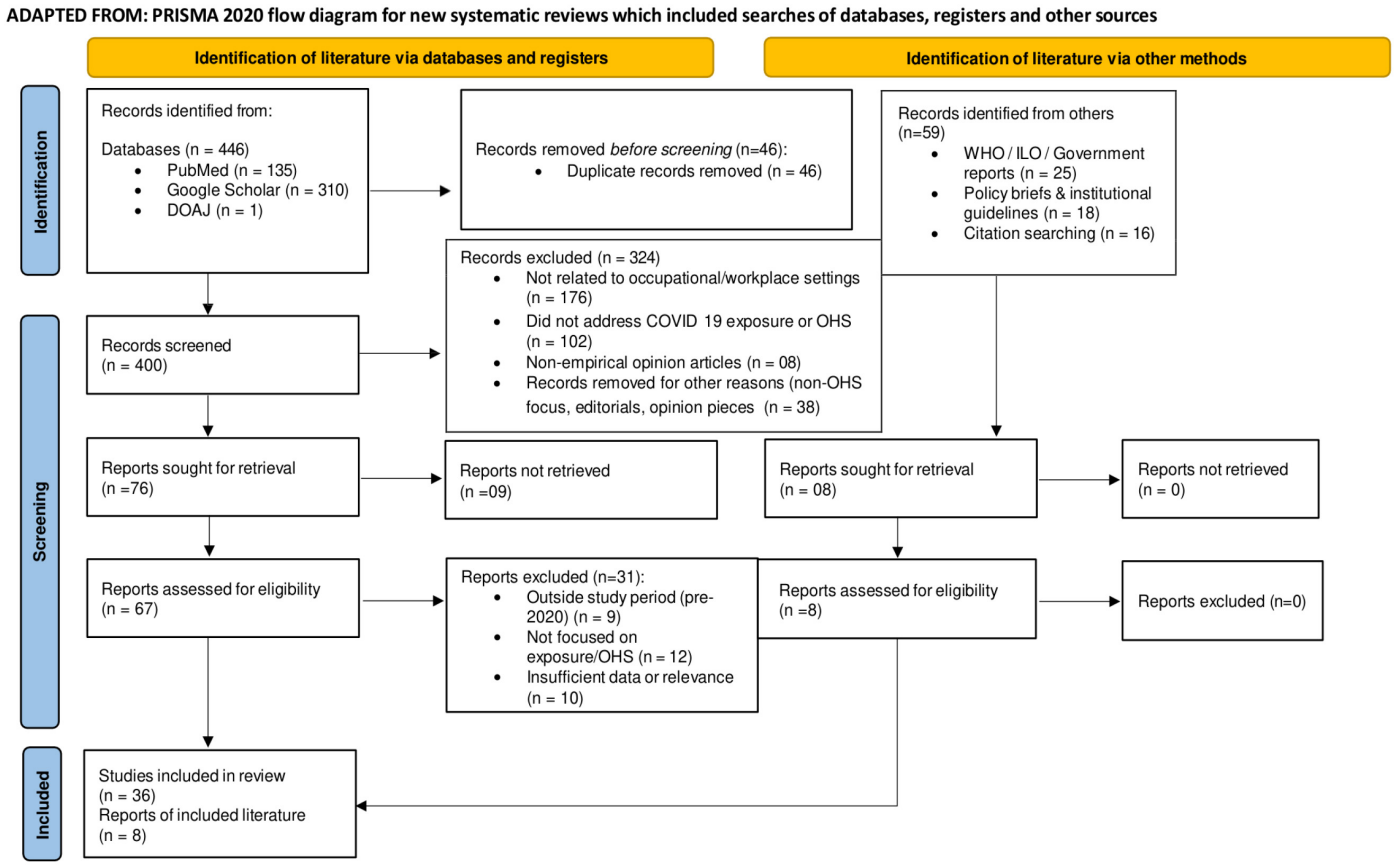
PRISMA flowchart of study methodology (21).

This systematic review was conducted in accordance with the Preferred Reporting Items for Systematic Reviews and Meta-Analyses (PRISMA) guidelines, to ensure transparency, reproducibility, and methodological rigor (21). This approach synthesized existing knowledge on the impact of the COVID-19 pandemic on OHS. Figure 1 illustrates the study methodology. Table 1 summarizes the key characteristics of the included studies.

**Table 1:** Summary of studies and literature on OHS, workplace impact, and COVID-19.

S. No.	Title of the study	References	Type of article	Country and area of the research	Study occupational group	Major outcome
1	Occupational health risk among healthcare workers during COVID-19 pandemic: actions to limit the risk	Casper (1)	Letter to Editor	Egypt	HCWs	Highlighted elevated occupational risk among HCWs and emphasized PPE, infection control, and administrative measures
2	COVID-19: Challenges, opportunities and lessons for occupational health	Zhang et al. (3)	Editorial	Global	All occupational groups	Highlighted lessons in surveillance, preparedness, and occupational health policy
3	COVID-19 outbreaks linked to workplaces	Luckhaupt et al. (4)	Surveillance study	USA	Multiple occupational sectors	Identified frequent workplace-linked outbreaks, especially in manufacturing, food processing, and healthcare
4	A systematic review of how remote work affects workplace stress and mental health	Guidarini and Hussaein (5)	Systematic review	Global	Remote workers	Found reduced infection risk but increased stress, isolation, and mental health concerns
5	Business sectors' initiatives on health and safety protocols and vaccination programs	Yutuc et al. (6)	Short Communication	Philippines	Corporate employees	Reported effective workplace vaccination and Infection Prevention and Control (IPC) protocols
6	Psychosocial impact of COVID-19 pandemic on healthcare workers in India	Chakma et al. (7)	Qualitative study	India	(HCWs)	Identified stress, fear, stigma, and need for psychological support
7	Changes in lifestyle, food choices, sleeping habits, and physical activities during COVID-19	Raheem et al. (8)	Cross-sectional study	India	General working population	Reported adverse lifestyle changes affecting worker health
8	Recognition of COVID-19 with occupational origin in Europe	Nys et al. (9)	Cross-sectional study	Europe	Workers across sectors	Demonstrated variation in recognition of COVID-19 as an occupational disease
9	Impact assessment of COVID-19 on Indian agriculture	Ahmad (10)	Review article	India	Agricultural workers	Showed livelihood disruption and occupational vulnerability
10	COVID-19 and healthcare workers: a rapid systematic review	Gross et al. (11)	Systematic review	Global	HCWs	Confirmed high infection risk and importance of PPE and vaccination
11	COVID-19 and the workplace: implications for future research	Kniffin et al. (12)	Narrative review	Global	All occupational groups	Provided framework for future workplace research
12	COVID-19: occupational health and safety for health workers	WHO (13)	Guideline	Global	HCWs	Provided standardized OHS and infection prevention guidance
13	Staff rostering and PPE to minimize transmission	Lim et al. (14)	Simulation study	Global	HCWs	Demonstrated effectiveness of split-team models
14	Guidance on preparing workplaces for COVID-19	OSHA (15)	Guideline	USA	All occupational groups	Provided workplace preparedness framework
15	WHO Coronavirus (COVID-19) Dashboard	WHO (16)	Surveillance database	Global	All occupational groups	Provided real-time global data on COVID-19 cases and deaths relevant to occupational exposure
16	India detects new COVID-19 sub-variants NB.1.8.1 and LF.7	India Today Web Desk (17)	News report	India	General workforce	Reported emerging variants and reinforced booster vaccination to reduce population and workplace risk
17	COVID-19: occupational health and safety for health workers	WHO (18)	Guideline	Global	HCWs	Provided standardized OHS and infection prevention guidance
18	Unemployment in the time of COVID-19	Blustein et al. (19)	Review	Global	Unemployed workers	Highlighted mental health and livelihood challenges
19	Burnout among healthcare workers during COVID-19 in India	Khasne et al. (20)	Survey study	India	HCWs	Reported high prevalence of burnout
20	Estimation of differential occupational risk of COVID-19	Zhang (24)	Analytical study	USA	Multiple occupations	Identified higher risk among frontline and essential workers
21	Psychological distress and burnout among healthcare workers in India	Menon et al. (25)	Cross-sectional study	India	HCWs	Reported high levels of burnout, anxiety, and depression
22	Reopening of schools during COVID-19 pandemic	Anand et al. (26)	Commentary	India	Teachers and school staff	Discussed occupational risks and policy dilemmas
23	Digital divide and access to online education	Jafar et al. (27)	Observational study	India	Educators and students	Highlighted occupational inequity due to limited digital access
24	Working from home during COVID-19 and its impact on stress and creativity	Jaiswal and Arun (28)	Empirical study	India	Remote workers	Found increased stress with variable impact on creativity
25	Hierarchy of controls	NIOSH (29)	Framework guideline	Global	All occupational groups	Described layered occupational risk control strategies
26	Analysis of hierarchy of controls in workplaces involving nanomaterials	Omari Shekaftik et al. (30)	Analytical study	Global	Laboratory and industrial workers	Reinforced hierarchy of controls for exposure reduction
27	Frontline HCWs' mental distress and hierarchy of controls	Zhang et al. (31)	Cross-sectional study	Global	HCWs	Found mental distress linked to inadequate controls
28	Organizational support and vaccination intention	Kobayashi et al. (32)	Cross-sectional study	Japan	Workers	Found higher vaccination intent with organizational support
29	Impact of COVID-19 on immunization programs	Lassi et al. (33)	Systematic review	Global	Healthcare systems	Reported disruption of routine immunization
30	COVID-19 vaccine challenges	Yarlagadda et al. (34)	Review	Global	Healthcare and public health workers	Identified logistical and acceptance barriers
31	COVID vaccine hesitancy in developing nations	Mallapaty (35)	Commentary	Global	General workforce	Highlighted growing vaccine hesitancy
32	COVID vaccines approval in less-affluent countries	Kozlov (36)	Reports	Global	General population	Reported higher acceptance in Low- and Middle-Income Countries (LMICs)
33	Preventive measures to be taken to contain the spread of Novel Coronavirus (COVID-19)	Govt. of India (37)	Government order	India	Government employees and public	Outlined administrative prevention measures
34	ILO response to COVID-19	Selberg (38)	Policy review	Global	Workers and labor sector	Emphasized labor rights and protections
35	Ayushman Bharat and COVID-19 impact	Gogoi et al. (39)	Review	India	Vulnerable populations	Highlighted role of health insurance in pandemic
36	SARS-CoV-2 antibody prevalence by industry	Gigot et al. (40)	Observational study	USA	Multiple industries	Showed industry-specific exposure risk
37	COVID-19, migration and livelihood in India	Bhagat et al. (41)	Review	India	Migrant workers	Identified severe livelihood disruption
38	COVID Katha: Multimedia guide for awareness	NCSTC (42)	Awareness initiative	India	General population	Enhanced public and worker awareness
39	Caring for healthcare warriors: mental health support	Jaisoorya et al. (43)	Guidance document	India	HCWs	Provided mental health support framework
40	Protecting workers: mitigating COVID-19 spread at workplace	OSHA (44)	Guideline	USA	All workers	Recommended layered prevention strategies
41	Establishing quarantine facilities during COVID-19	Garg et al. (45)	Research Article	India	Healthcare and administrative staff	Identified operational challenges
42	Strategy for COVID-19 vaccination in India: the country with the second highest population and number of cases	Kumar et al. (46)	Policy analysis	India	Healthcare and general population	Outlined India's large-scale vaccination strategy
43	Impact of COVID-19 on migrant workers in India	GCAP (47)	Policy report	India	Migrant workers	Documented job loss and social insecurity

### 2.1 Search strategy

A comprehensive literature search was conducted across multiple electronic databases: PubMed, Google Scholar, Directory of Open Access Journals (DOAJ) and ResearchGate. In addition, gray literature (government reports, WHO/ILO documents, policy briefs, and institutional guidelines) was manually searched.

### 2.2 Search keywords

The following keywords were combined using Boolean operators “AND,” “OR,” and “NOT.”

1. OHS
2. COVID-19 Pandemic
3. SARS-CoV-2 infection and occupation
4. Workplace transmission
5. Occupational exposure
6. Infection control
7. Personal Protective Equipment
8. Pandemic preparedness
9. Telework and remote work
10. Mental health impact of COVID-19
11. Long COVID effects
12. Employee health
13. Public health policy
14. Workplace guidelines
15. Health Risk Assessment
16. Occupational Hazards
17. Psychological support

### 2.3 Search string

Search terms were structured into three domains:

1. COVID-19 and SARS-CoV-2 infection,
2. occupational health and workplace exposure, and
3. workplace mitigation strategies and health impacts.

Detailed search strings for each database are provided in Supplementary File S1.

### 2.4 Eligibility criteria

Inclusion criteria:

1. Articles published between January 2020 and December 2024;
2. Peer-reviewed original research- observational studies, reviews, systematic reviews, and policy/guideline documents, letter to editor, surveillance study, short communication, Simulation study, Commentary, News report;
3. Studies focusing on occupational exposure, workplace transmission, OHS guidelines, or workforce health during COVID-19;
4. Publications in English language only.

Exclusion criteria:

1. Studies unrelated to occupational or workplace settings
2. Opinion pieces without empirical or policy relevance

### 2.5 Study selection process

All retrieved records were initially screened by title and abstract for relevance by two independent reviewers. Duplicates were removed and full texts were retrieved. Full-text articles were assessed for eligibility based on eligibility criteria. Disagreements during selection were resolved through discussion among authors.

### 2.6 Data extraction

Relevant data were extracted using a form, including: Study setting and occupational sector; type of occupational exposure; COVID-19 transmission patterns; preventive and control measures implemented; changes in OHS policies and guidelines; key outcomes and conclusions. Extracted information was synthesized narratively to identify recurring themes and policy-relevant insights.

### 2.7 Quality assessment

The methodological quality of the included observational/surveillance/analytical studies was assessed using the Newcastle–Ottawa Scale (NOS) (22). The scale evaluates studies based on three domains: selection of study participants, comparability of study groups, and outcome/exposure assessment. Studies were scored out of a maximum of 10 points, and were categorized as high (8–10), moderate (5–7), or low (< 5) quality.

The methodological quality of included systematic reviews was assessed using the JBI Critical Appraisal Checklist for Systematic Reviews and Research Syntheses (23). This tool evaluates the clarity of the review question, appropriateness of inclusion criteria, search strategy, critical appraisal process, and methods of data synthesis to ensure methodological rigor.

## 3 Results

A total of 43 articles from the scholarly and gray literature met the eligibility criteria and were included in the full-text review. These comprised observational studies, cross-sectional studies, analytical studies, surveillance reports, and policy or guideline documents addressing OHS during the COVID-19 pandemic. Overall, the scope and methodological quality of the included studies varied, reflecting differences in study design, occupational settings, and geographic coverage. The included literature collectively provided insight into occupational exposure patterns, workplace mitigation strategies, and policy responses implemented during the COVID-19 pandemic. The quality assessment scores of 14 observational studies are given in Table 2 using NOS. The quality assessment scores of 03 systematic review studies are given in Table 3 using JBI Critical Appraisal Checklist for Systematic Reviews and Research Syntheses.

**Table 2:** Quality assessment of included observational studies using the Newcastle–Ottawa Scale (NOS).

S. No.	References	Study design	Selection (5)	Comparability (2)	Outcome (3)	Total score (10)	Quality rating
1	Luckhaupt et al. (4)	Surveillance/Observational	4	2	3	9	High
2	Raheem et al. (8)	Cross-sectional	3	1	2	6	Moderate
3	Nys et al. (9)	Observational comparative	4	2	2	8	High
4	WHO Dashboard (16)	Surveillance data	3	1	2	6	Moderate
5	Khasne et al. (20)	Cross-sectional survey	4	1	2	7	Moderate
6	Zhang et al. (24)	Analytical observational	4	2	3	9	High
7	Menon et al. (25)	Cross-sectional	4	1	2	7	Moderate
8	Jafar et al. (27)	Observational	3	1	2	6	Moderate
9	Jaiswal et al. (28)	Analytical study	4	1	2	7	Moderate
10	Omari Shekaftik et al. (30)	Analytical/workplace risk analysis	4	2	2	8	High
11	Zhang et al. (31)	Cross-sectional	4	1	2	7	Moderate
12	Kobayashi et al.,2021^32^	Cross-sectional	4	2	2	8	High
13	Gigot et al. (40)	Observational seroprevalence study	5	2	3	10	High
14	Garg et al. (45)	Field observational study	3	1	2	6	Moderate

**Table 3:** Quality assessment of included systematic studies using the JBI checklist.

S. No.	References	Q1	Q2	Q3	Q4	Q5	Q6	Q7	Q8	Q9	Q10	Q11	Overall result
1	Guidarini et al. (5)	Yes	Yes	Yes	Yes	Yes	No	Yes	Yes	Unclear	Yes	Yes	Include
2	Gross et al. (11)	Yes	Yes	Yes	Yes	Yes	No	Yes	No	No	Yes	Yes	Include
3	Lassi et al. (33)	Yes	Yes	Yes	Yes	Yes	Yes	Yes	Yes	Yes	Yes	Yes	Include

### 3.1 Impact of COVID-19 on various occupational sectors

The Occupational Safety and Health Administration (OSHA) classified occupations into four distinct risk categories—very high, high, medium, and lower risk—based on the likelihood of exposure to SARS-CoV-2 (15). The effects of the COVID-19 pandemic varied considerably across occupational sectors, influenced by differences in exposure intensity, work environment, and the ability to adopt infection prevention strategies. HCWs bore the greatest burden of infection risk, attributable to continuous patient care, involvement in aerosol-producing procedures, and staffing constraints, which collectively contributed to heightened psychological strain and burnout (11, 18). Workers engaged in essential services—such as law enforcement, sanitation, and transportation—also encountered increased vulnerability owing to ongoing interaction with the public (24). Conversely, employees in sectors like IT and administrative services largely shifted to remote working arrangements, which lowered the likelihood of viral transmission but were associated with adverse mental health outcomes, social disconnection, and challenges in maintaining work–life balance (5, 25). In industries such as manufacturing, retail, and the informal sector, the pandemic resulted in substantial work interruptions, financial instability, and inconsistent application of occupational safety measures. The education sector experienced extended closures, necessitating a transition to digital learning platforms that were marked by unequal access and heightened occupational stress among teaching staff (26). A sector-wise overview of the occupational impact of COVID-19 is summarized in Table 4.

**Table 4:** Impact of COVID-19 on various occupational sectors.

Occupational sectors	Exposure risk (based on OSHA class)	Key impacts	References
Healthcare	Very high risk	High infection rates, burnout, mental distress	Gross et al. (11); Chakma et al. (7)
Essential services	High risk	Increased exposure, variable PPE access	Zhang (24)
Manufacturing and industry	Medium-High risk	Shutdowns, economic loss, infection clusters	Luckhaupt et al. (4)
Education	Medium risk	Online transition, psychological strain	Anand et al. (26); Jafar et al. (27)
IT and Corporate	Low risk	Reduced infection, increased stress, isolation	Jaiswal and Arun (28)
Retail and informal sector	Medium-High risk	Job insecurity, inconsistent safety measures	Kniffin et al. (12)

### 3.2 Strategies implemented to prevent transmission of COVID-19 in occupational settings

The National Institute for Occupational Safety and Health (NIOSH), United States of America (USA) implemented the workplace transmission control strategies using the hierarchy of controls framework. Engineering controls such as improved ventilation, physical barriers, and isolation rooms were prioritized in high-risk settings. Administrative measures included staggered shifts, remote work policies, screening, testing, and contact tracing. Safe work practices focused on hygiene, surface disinfection, and physical distancing. PPE was widely adopted when higher-level controls were insufficient. These layered interventions effectively reduced workplace outbreaks and secondary community transmission (12, 30, 31). Table 5 represents the classification of work exposure levels with their control strategies.

**Table 5:** Key OHS initiatives implemented in India during COVID-19.

S.No.	Initiative	Target group	Purpose	Reference
1	Workplace SOPs	All sectors	Infection control	GoI (37)
2	Aarogya Setu App	Employees	Contact tracing	GoI (42)
3	Ayushman Bharat	Informal workers	Financial protection	Gogoi et al. (39)
4	Migrant welfare schemes	Migrant workers	Livelihood support	Bhagat et al. (41)
5	Mental health framework	HCWs	Psychological support	NIMHANS (43)

#### 3.2.1 Risk-based classification of workplace exposure and control measures

It was seen from the reviewed articles that workplace exposure to SARS-CoV-2 was broadly categorized into high, medium, and lower risk levels, with corresponding control measures guided by the hierarchy of controls framework (12). High-risk occupations, including HCWs paramedical staff, laboratory personnel, and morgue workers, involve direct exposure to infected individuals and aerosol-generating procedures. These settings require robust engineering controls such as adequate ventilation and airborne infection isolation rooms, along with strict administrative measures, safe work practices, and comprehensive use of PPE to minimize exposure (12). Medium-risk occupations, such as teachers, retail workers, and public transport employees, involve frequent public interaction. Control measures include physical barriers, symptom screening, reduced contact strategies, routine cleaning and disinfection, and task-specific use of PPE (12). Lower-risk occupations, including IT professionals and office workers, involve minimal exposure risk. Preventive measures primarily focus on adherence to public health guidance, effective communication, general hygiene practices, and routine workplace safety protocols, with no additional PPE required beyond standard use (12). Detailed tabular information is provided in Supplementary File S2.

### 3.3 Strategic role of COVID-19 vaccination in protecting workplace health

Vaccination emerged as a critical preventive strategy in occupational health, significantly reducing severe disease, absenteeism, and workplace outbreaks (32). Prioritization of healthcare and frontline workers ensured workforce continuity and enhanced organizational resilience. Despite logistical and vaccine hesitancy challenges, vaccination programs contributed substantially to stabilizing occupational health systems and restoring economic productivity (33, 34). A survey involving 45,000 participants, conducted across 12 countries from June 2020 to January 2021, revealed that individuals in LMICs showed less hesitation toward receiving the vaccine compared to those in countries such as the US and Russia (35). Overall, 80% of participants surveyed in Asia, Africa, and South America expressed willingness to receive the vaccine, compared to only 30% in Russia and 65% in the US indicated the same readiness (36).

### 3.4 OHS initiatives by India during the COVID-19 pandemic

India implemented comprehensive OHS initiatives integrating public health and workplace safety. National guidelines mandated thermal screening, staggered attendance, testing, and contact tracing. Digital tools such as the Aarogya Setu app supported workplace surveillance (15, 18, 37). Vaccination campaigns prioritized high-risk occupational groups, while welfare schemes addressed the needs of migrant and informal workers. Mental health support frameworks for HCWs further expanded the scope of occupational health beyond physical hazards.

During the COVID-19 pandemic, global and national initiatives were implemented to strengthen OHS for workers, particularly in vulnerable sectors. The WHO and ILO issued comprehensive protocols for maintaining occupational safety, promoting the use of PPE, physical distancing, and sanitation measures (38). These global efforts were complemented by policies at the national level in India aimed at protecting workers' health, such as the Ayushman Bharat scheme. This health insurance program was expanded to include COVID-19 treatment, offering free coverage to millions of vulnerable citizens, including those in informal sectors, thereby easing their financial burden during the pandemic (39). Table 5 represents the key OHS initiatives.

To effectively manage infection control and prevention in workplaces throughout the COVID-19 pandemic, various guidelines and policies were issued by ministries, the Department of Science and Technology, and the Indian Council of Medical Research. These measures were aimed at ensuring the safety of employees while maintaining operational continuity. Some of these key guidelines included:

1. Thermal screening: one of the primary measures implemented was thermal screening at workplace entry points. Employees were required to undergo mandatory temperature screening before entering the premises, with those showing elevated temperatures being advised to seek medical consultation or self-isolate (40).
2. Rotational attendance: to minimize the risk of overcrowding and maintain physical distancing, workplaces adopted rotational attendance policies. Employees were divided into different groups, with each group attending the office on alternate days or shifts, thereby minimizing the number of people present at any given time. Adequate rest during their shift was provided to the workers (3).
3. Leave policies for infected employees: specific leave policies were given to employees who were tested positive for COVID-19. These policies ensured that infected employees could take the necessary time off to recover without the fear of losing their jobs or income. Paid sick leave and extended medical leave options were typically provided (41).
4. Testing and contact tracing: if an employee was found to be infected, prompt testing of close contacts, including colleagues, was mandated. This was crucial for early detection and prevention of further spread within the workplace. Some organizations also collaborated with local health authorities to facilitate rapid testing for employees (40).
5. Aarogya Setu App: the Aarogya Setu app served a key function in tracking employee health and enabling workplace reporting mechanisms. Employees were encouraged or required to install the app, which helped in contact tracing and provided real-time information about potential exposure to COVID-19. The app's integration into workplace protocols enabled employers to monitor the health status of their workforce and take timely action if an infection was reported (37, 40).

## 4 Discussion

This systematic review highlights the profound and multidimensional impact of the COVID-19 pandemic on OHS, reinforcing occupation as a critical determinant of SARS-CoV-2 exposure, disease burden, and health outcomes. By synthesizing findings from previously published studies conducted in India and across different global settings, this review provides a comprehensive perspective on occupational exposure patterns, workplace transmission dynamics, and sector-specific vulnerabilities observed during the COVID-19 pandemic. To the best of our knowledge, this review is among the first to systematically synthesize structured evidence on OHS in the context of COVID-19 by integrating findings from both Indian and global studies. The findings highlight how differential occupational risks, workplace transmission dynamics, and sector-specific vulnerabilities influenced both the spread of COVID-19 and the evolution of OHS frameworks. This review adopts a comprehensive perspective by examining: the impact of COVID-19 on occupational exposure across different sectors, workplace strategies implemented to prevent transmission, and OHS initiatives implemented in India during the COVID-19 pandemic.

### 4.1 Occupational risk stratification and sector-specific impact

The results demonstrated that COVID-19 disproportionately affected occupational groups based on exposure intensity, work environment, and feasibility of implementing preventive controls. HCWs consistently faced the highest occupational risk, owing to sustained exposure to infected individuals, aerosol-generating procedures, and workforce shortages, corroborating findings from Indian and global studies (9, 18–20). Elevated infection rates among healthcare personnel were compounded by psychological distress, burnout, fear of infecting family members, and social stigma, highlighting the dual physical and mental health burden associated with frontline roles (7, 16, 20). These observations are consistent with multiple Indian and international studies reporting higher infection rates among frontline workers compared with the general population, emphasizing the critical role of occupational exposure in shaping pandemic transmission patterns.

Beyond healthcare, essential service workers—including transport, sanitation, and public safety personnel—were exposed to heightened risks due to continuous public interaction and limited opportunities for remote work (24). Manufacturing, food processing, and informal sectors experienced notable workplace-linked outbreaks, particularly in crowded settings with inadequate ventilation and inconsistent adherence to safety protocols, as observed in surveillance studies from India and USA (4, 29). These findings highlight structural inequalities in occupational risk distribution, where workers engaged in public-facing or manual occupations experienced disproportionately higher exposure risks compared with those in remote-enabled sectors. In contrast, remote-enabled sectors such as IT and administrative services demonstrated reduced infection risk but reported increased mental stress, social isolation, and work–life imbalance, emphasizing that lower biological risk did not equate to reduced occupational strain (5, 28). This divergence illustrates that occupational health impacts during the pandemic extended beyond infection risk to include significant psychosocial consequences.

The OSHA-based occupational risk classification adopted across studies proved instrumental in contextualizing exposure levels and guiding tailored workplace interventions (15). This stratified approach reinforces the need for risk-based OHS planning rather than uniform, one-size-fits-all measures, particularly in heterogeneous workforces such as those seen in India.

### 4.2 Effectiveness of hierarchy-based workplace control measures

The implementation of layered workplace interventions, guided by the hierarchy of controls framework, emerged as a cornerstone of occupational COVID-19 mitigation from Indian settings. Engineering controls—such as improved ventilation, isolation rooms, and physical barriers—were particularly effective in high-risk healthcare and industrial settings, aligning with WHO, OSHA, and NIOSH recommendations (12, 18). Globally it was seen that administrative controls, including staggered shifts, telework, symptom screening, testing, and contact tracing, significantly reduced workplace density and enabled early identification of infections, thereby limiting outbreak amplification (15, 44). Evidence from several observational and workplace surveillance studies further demonstrated that workplaces implementing multiple layers of preventive measures reported lower transmission rates compared with those relying solely on individual-level protective practices.

Garg et al. (45) reported that safe work practices, including hand hygiene, surface disinfection, and physical distancing, complemented higher-level controls, while PPE served as a critical last line of defense, especially in settings where exposure could not be eliminated (9). Evidence from healthcare and emergency response settings demonstrated that inadequate implementation of these controls was associated with increased infection risk and psychological distress, reinforcing the interdependence of physical safety and mental wellbeing (31).

Collectively, these findings highlight that the success of workplace transmission prevention relied not on isolated interventions but on integrated, multi-layered strategies tailored to occupational risk profiles. The hierarchy of controls—comprising elimination, substitution, engineering controls, administrative controls, and personal protective equipment (PPE)—should be systematically embedded in workplace preparedness planning. However, implementation varied considerably across sectors, particularly within informal workplaces and small enterprises where infrastructural limitations and weak regulatory enforcement reduced the effectiveness of recommended safety measures.

### 4.3 Vaccination as a pillar of occupational health protection

Vaccination emerged as a transformative intervention in protecting workforce health and ensuring organizational resilience. Prioritization of healthcare and frontline workers during early vaccination phases significantly reduced severe disease, absenteeism, and workforce attrition, supporting continuity of essential services (32, 46). Organizational support was shown to positively influence vaccine acceptance among employees, underscoring the role of employers in promoting public health interventions within occupational settings (32). These findings align with global evidence demonstrated that workplace vaccination programs contributed significantly to reducing occupational outbreaks and maintaining workforce stability during later phases of the pandemic.

Despite logistical challenges and vaccine hesitancy, particularly during the early rollout phases, studies indicated comparatively higher acceptance rates in low- and middle-income countries (LMICs), including India, than in several high-income nations (35, 36). In India, large-scale vaccination drives targeting healthcare personnel, frontline workers, and essential service providers played a crucial role in stabilizing workforce capacity and facilitating the gradual resumption of economic activities. However, disruptions to routine immunization services and the spread of misinformation posed additional challenges, underscoring the need for sustained risk communication and trust-building measures (33, 34).

Risk communication emerged as a critical component of the pandemic response, playing a central role in addressing misinformation, promoting adherence to preventive measures, and enhancing vaccine acceptance. Effective communication strategies fostered trust among employers, employees, and public health authorities, thereby improving compliance with workplace safety protocols. These observations reinforce that vaccination is not merely a biomedical intervention but an integral component of comprehensive occupational health policy. Accordingly, risk communication should be institutionalized as a core component of occupational health systems to strengthen preparedness and response to future public health emergencies.

### 4.4 Transformation of OHS frameworks and India's policy response

Beyond immediate infection control, the pandemic catalyzed a fundamental shift in OHS paradigms. While traditional OHS frameworks have largely focused on physical and chemical hazards, the COVID-19 pandemic highlighted the critical role of Occupational Hygiene/Industrial Hygiene (OH/IH) in managing biological risks. Strengthening this discipline within India's occupational health system is essential for advancing evidence-based prevention and enhancing preparedness for future infectious disease threats (3, 29). The recognition of COVID-19 as an occupationally acquired disease across multiple sectors strengthened the call for inclusive and adaptive OHS policies (9). This shift was particularly evident in India, where public health authorities and labor institutions implemented coordinated workplace policies aimed at protecting both formal and informal sector workers.

In India, national OHS initiatives reflected this transition through coordinated workplace Standard Operating Procedures (SOPs), thermal screening, rotational attendance, testing protocols, and the deployment of digital surveillance tools such as the Aarogya Setu app (37, 42). Social protection measures, including expansion of Ayushman Bharat coverage and targeted migrant welfare schemes, addressed the disproportionate burden borne by informal and migrant workers, who faced job loss, mobility restrictions, and limited access to healthcare (39, 41, 47).

Furthermore, mental health support frameworks for HCWs acknowledged the psychological toll of pandemic response, marking a critical advancement in holistic occupational health planning (20, 25, 43). Nevertheless, the pandemic also revealed persistent gaps in occupational health coverage, particularly for informal workers who constitute a substantial proportion of India's labor force and often lack structured workplace safety protections. These integrated efforts illustrate India's progression toward a more resilient and equitable OHS system capable of responding to future public health emergencies.

### 4.5 Structural gaps identified

There are several limitations in Indian studies that highlight gaps in evidence regarding occupational exposure and workplace- related impacts during the COVID-19 pandemic. For instance, the study by Chakma et al. (7) primarily examined the psychosocial impact of the pandemic on HCWs and they found gaps in mental health preparedness, indicating the need for broader occupational health investigations, including systematic exposure assessment and risk evaluation led by OH/IH professionals. The research by Raheem et al. (8) focused on lifestyle and behavioral changes during the pandemic across three Indian states; however, the study did not specifically evaluate occupational exposure or workplace safety interventions, highlighting a gap in understanding workplace-related determinants of health during the pandemic. The work of Bhagat et al. (41) mainly addressed migration patterns and policy implications during the pandemic, with limited emphasis on occupational health risks or workplace safety measures, underscoring the need for more focused research on occupational exposure among vulnerable worker groups. Gross et al. identified substantial gaps in occupational health evidence, including lack of standardized exposure assessment, and limited evaluation of intervention effectiveness. These findings highlight the need for robust occupational research strategies, including longitudinal studies and standardized surveillance systems, to better inform workplace health policies (11). Khasne et al. is limited by its cross-sectional design, self-reported data, and lack of longitudinal assessment, restricting causal inference and generalizability. These gaps emphasize the need for stronger occupational research strategies, including longitudinal and multi-center studies, to better understand and address healthcare worker burnout (20). These requirements align closely with the established competencies of OH/IH particularly in hazard identification, exposure assessment, and implementation of preventive control strategies.

## 5 Implications for future occupational health preparedness

The findings of this review emphasize that occupational health must be positioned at the intersection of public health, labor policy, and social protection. Future preparedness strategies should prioritize risk-based workplace controls guided by the hierarchy of controls, ensuring that elimination and engineering controls are implemented wherever feasible, followed by administrative strategies and appropriate use of PPE.

Strengthening occupational health surveillance systems and integrating them with national public health frameworks will be essential for early detection and response to emerging threats. In addition, the expansion of OH/IH capacity will play a critical role in systematic exposure assessment and prevention-focused workplace interventions.

Risk communication strategies must also be strengthened to address misinformation, improve compliance with preventive measures, and enhance trust in workplace health policies. Furthermore, targeted interventions are needed to protect vulnerable worker populations, particularly those in informal sectors, through inclusive policy frameworks and improved access to occupational health services.

Overall, a prevention-oriented, multi-sectoral approach will be critical for building resilient occupational health systems capable of responding effectively to future pandemics.

## 6 Conclusion

The COVID-19 pandemic profoundly disrupted OHS systems worldwide, with frontline HCWs and essential service personnel experiencing disproportionately higher risks of infection and adverse health outcomes. This review highlights workplace exposure as a critical determinant of COVID-19 transmission, underscoring occupation as an important risk factor shaping infection control policies and prevention strategies. The differential impact observed across occupational sectors emphasized the necessity of risk-based, sector-specific interventions, with high-risk essential services requiring more stringent control measures compared to lower-risk non-essential sectors. Globally, the pandemic catalyzed the evolution of robust OHS frameworks, integrating engineering and administrative controls, safe work practices, appropriate use of personal protective equipment, and vaccination strategies to mitigate workplace transmission. In India, comprehensive public health and occupational initiatives—including prioritized vaccination of high-risk workers, establishment of dedicated COVID-19 care facilities, digital surveillance tools, and workplace safety guidelines—played a crucial role in protecting the workforce and maintaining essential services. The recent detection of emerging SARS-CoV-2 subvariants in India reinforces the need for sustained vigilance and adaptive occupational health strategies in the face of evolving viral threats. Lessons learned from the COVID-19 pandemic underscore the importance of developing resilient, evidence-based, and inclusive OHS frameworks capable of addressing future public health emergencies. Strengthening occupational surveillance, integrating mental health support, and safeguarding vulnerable worker populations will be essential to ensuring workforce preparedness and resilience in an era of increasing global health and environmental challenges.

## Data Availability

The original contributions presented in the study are included in the article/Supplementary material, further inquiries can be directed to the corresponding authors.
