# Supplementary material for: Occupational health and safety in the wake of COVID-19: insights from India's workforce
Source: Front Public Health. 2026 Jun 9;14:1807940. doi: 10.3389/fpubh.2026.1807940 (PMC13287014; doi:10.3389/fpubh.2026.1807940)
Supplement: Supplementary file 1 [file Supplementary_file_1.pdf]

Classification of Workplace Exposure and Corresponding Control Measures [12]

Work Exposure	Type of Job	Workplace Controls			
		Engineering controls	Administrative controls	Safe work practices	Personal Protective Equipment (PPE)
High Risk	HCWs (doctors, nurses, paramedic staff, laboratory personnel) and morgue workers	Implemented and maintained adequate ventilation systems in medical facilities, with Centers for Disease Control and Prevention (CDC) recommending placing individuals with confirmed or suspected COVID-19 in airborne infection isolation rooms (AIIR) when	Followed existing guidelines and facility standards in healthcare settings to identify and isolate infected individuals and protect workers. Developed policies to reduce occupational risk by grouping infected patients in designated zones, posted signs requesting symptom	To facilitate effective field decontamination, emergency responders and other essential staff working remotely were equipped with alcohol-based hand sanitizers containing at least 60% alcohol. Using Biomedical waste management practices; and antiseptic measures to clear workplace surfaces	Workers identified as having high or very high exposure risk were provided with comprehensive personal protective equipment, comprising gloves, gowns, goggles, face shields, and either respirators or face masks, based on their specific job responsibilities.

		feasible. Used isolation rooms or autopsy suites for generating respiratory aerosols on COVID-19 patients or deceased individuals, following Biosafety Level 3 precautions for handling specimens.	reporting, offered enhanced medical monitoring during outbreaks, provided job-specific training on COVID-19 prevention, and ensured psychological and behavioral support for employees.		
Medium	Teachers, other school & university staff, Retail workers, public transportation employees, restaurant staff	To reduce exposure, physical barriers such as transparent sneeze guards were implemented in feasible locations.	Offered face masks to ill employees and customers.<br><br>To reduce transmission risk, customers were educated about COVID-19 symptoms and urged to	Cleaning of desks with detergents and disinfectants.	Workers with medium exposure risk needed to don gloves, gowns, face masks, and/or eye and face protection, with personal protective equipment determined by

			limit contact with workers if experiencing illness.<br><br>Implemented measures to reduce in-person interaction and communicated the availability of medical monitoring and worker health resources.		task requirements, employer-assessed occupational risks and duties-related exposures
Lower Risk	IT professionals, researchers (Non-Medical), delivery drivers, Security personnel, factory and warehouse workers, and office workers.	Employees classified as lower-risk did not require supplemental engineering controls. Employers were expected to maintain and ensure the proper functioning of any existing	Monitored public health communications regarding COVID-19 recommendations and ensured workers had access to this information. Collaborated with workers to identify effective methods for conveying important	Nil	Workers in the low exposure category were not instructed to utilize supplementary personal protective equipment. Workers were advised to continue using the PPE they ordinarily used for their other job tasks

		controls designed to mitigate other occupational hazards.	COVID-19 information.		
